# Surgical outcomes in gastroenterological surgery in Japan: Report of the National Clinical Database 2011‐2018

**DOI:** 10.1002/ags3.12324

**Published:** 2020-03-20

**Authors:** Yoshihiro Kakeji, Arata Takahashi, Hiroshi Hasegawa, Hideki Ueno, Susumu Eguchi, Itaru Endo, Akira Sasaki, Shuji Takiguchi, Hiroya Takeuchi, Masaji Hashimoto, Akihiko Horiguchi, Tadahiko Masaki, Shigeru Marubashi, Kazuhiro Yoshida, Mitsukazu Gotoh, Hiroyuki Konno, Hiroyuki Yamamoto, Hiroaki Miyata, Yasuyuki Seto, Yuko Kitagawa, National Clinical Database

**Affiliations:** ^1^ The Japanese Society of Gastroenterological Surgery Tokyo Japan; ^2^ Department of Health Policy and Management School of Medicine Keio University Tokyo Japan; ^3^ Department of Healthcare Quality Assessment Graduate School of Medicine The University of Tokyo Tokyo Japan

**Keywords:** gastroenterological surgery, NCD, surgical outcome

## Abstract

The National Clinical Database (NCD) of Japan grew rapidly, harvesting over 11 million cases of data between 2011 and 2018 from more than 5000 facilities. This is the Report of the NCD based upon gastrointestinal surgery information in 4 420 175 cases from 2011 to 2018. More than 70% of all gastrointestinal surgeries were performed at certified institutions, and the percentage of surgeries performed at certified institutions was particularly high for the esophagus (93.8% in 2018), liver (89.4%), pancreas (91.3%), and spleen (86.9%). Also, more than 70% of the surgeries were performed with the participation of the board‐certified surgeon. As the patients have been getting older, the morbidities have been increasing. However, the mortalities have been kept at a low level. The rates of endoscopic surgery have been increasing year by year, especially high in low anterior resection (67.0%) and esophagectomy (61.0%). Nationwide, this database is surely expecting to ensure the quality of board certification system and surgical outcomes in gastroenterological surgery.

## Graphical abstract

This is the Report of the National Clinical Database (NCD) based upon gastrointestinal surgery information in 4 420 175 cases from 2011 to 2018. Nationwide this database is surely expecting to ensure the quality of the board certification system and surgical outcomes in gastroenterological surgery.

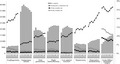

## 1 INTRODUCTION

The Japanese National Clinical Database (NCD), which started its data registration in 2011, has grown into a large nationwide database covering more than 95% of the surgeries performed by regular surgeons in Japan.^1^ As of the end of December 2019, 5276 facilities have enrolled in the NCD, and about 1 500 000 cases have been registered every year.^2^

In the gastroenterological section of the NCD, the Japanese Society of Gastroenterological Surgery (JSGS) selected 115 gastrointestinal operative procedures as important for the board certification system, and eight main procedures as especially important in terms of medical standards for improvement of surgical quality. All surgical cases are registered in the NCD with input of postoperative complications for the 115 procedures, and with detailed input such as comorbidities and morbidities for the eight main procedures; namely, esophagectomy, partial/total gastrectomy, right hemicolectomy, low anterior resection, hepatectomy, pancreaticoduodenectomy, and surgery for acute diffuse peritonitis.^3^, ^4^ Risk models of mortality^5^, ^6^, ^7^, ^8^, ^9^, ^10^, ^11^, ^12^ and morbidity^13^, ^14^, ^15^, ^16^, ^17^, ^18^, ^19^, ^20^ for these eight main procedures have been reported, which were all the first‐risk stratification studies based on a Japanese nationwide web‐based database. The risk calculator has been implemented using the risk models, which enables predictions of morbidity and mortality of patients after inputting the preoperative data.^21^ To secure the reliability of data collected from the gastroenterological section of the NCD, the JSGS started data verification activity in 2016 and found a high accuracy of data entry.^22^

Following the previous annual reports,^3^, ^4^ the Report of the NCD 2011‐2018 is based upon gastrointestinal surgery information in 4 420 175 cases of surgery performed and recorded from 2011 to 2018. It is our pleasure that this report would reflect the real world of Japanese surgical outcomes of gastroenterological surgery.

## 2 SUBJECTS AND METHOD

As previously reported,^3^, ^4^ the subjects were patients whose surgical data were recorded in the NCD, and who underwent one or more of the 115 surgical procedures stipulated by the “Training Curriculum for Board Certified Surgeons in Gastroenterology,“ using the “New Classification of Surgical Difficulty.” The board certification system of the JSGS consists of board‐certified training institutions and board‐certified surgeons in gastroenterological surgery.^23^ Requirements for board‐certified training institutions are 600 or more gastroenterological operations determined by the certified committee (more than 120 of which are essential major surgery) in the last 3 years. And board‐certified surgeons are required the experience performing 450 or more gastroenterological operations, and gastroenterological surgical training for more than 5 years according to the training curriculum in a board‐certified training institution authorized by the JSGS. We targeted data from 2011 to 2018, adding data of complications to data already reported in the Annual Report 2011‐2017. Complications included surgical site infection (SSI), wound dehiscence, anastomotic leakage, pancreatic fistula, bile leakage, pneumonia, unplanned intubation, pulmonary embolism, ventilator‐assisted respiration longer than 48 hours, progressive renal insufficiency, acute renal failure, urinary tract infection, cerebrovascular accident with neurological deficit, coma longer than 24 hours, peripheral nerve injury, cardiac arrest requiring cardiopulmonary resuscitation, myocardial infarction, bleeding complications defined by transfusions in excess of one unit of blood, deep venous thrombosis, and sepsis. Postoperative complications were categorized into six grades according to the Clavien–Dindo (C–D) classification.^24^ In this study, grade III (complications requiring intervention) or higher complications were defined as severe complications. Furthermore, we separated and studied the eight main operative methods that we deemed important in terms of medical standards among these 115 procedures.

We clarified the number of surgical cases and the mortality rates related to the selected 115 gastrointestinal operative procedures, and also the eight main operative procedures from 2011 to 2018. We also comparatively studied patient sex, age groups, institution groups, and the percentage contribution of certified surgeons related to the eight main operative procedures.

The following points need to be considered in the interpretation of the data reported here: (a) since a maximum of eight operative procedures can be recorded per each case in the NCD, the total number of surgeries in “Results of the 115 gastrointestinal surgical procedures for board certification system” is not the actual total number of surgical cases; (b) cases with errors in patient age, sex, and postoperative 30‐day status were excluded; (c) cases in which several operative methods were performed simultaneously were tallied per all operative methods; (d) postoperative 30‐day mortality included all cases of mortality within 30 days of surgery regardless of pre‐ or post‐discharge status. The calculation of operative mortality included all patients who died during the index hospitalization, including hospital stays up to 90 days, and any patient who died after hospital discharge within 30 days of the operation date.

## 3 RESULTS

### 3.1 The 115 selected gastrointestinal operative procedures in the “Training Curriculum for Board Certified Surgeons in Gastroenterology“

The total number of cases represented by the 115 selected gastrointestinal surgical procedures, recorded in the NCD between 1 January and 31 December 2018 was 601 754. Based on organ involvement, 9286 cases involved the esophagus (1.5%); 65 152 cases, the stomach and duodenum (10.8%); 236 496 cases, the small intestine and colon (39.3%); 56 162 cases, the rectum and anus (9.3%); 26 531 cases, the liver (4.4%); 139 844 cases, the gall bladder (23.2%); 19 152 cases, the pancreas (3.2%); 2544 cases, the spleen (0.4%); and 46 587 cases, other organs (7.7%) (Table 1). The increase of cases, especially with malignant colorectal diseases, was remarkable, while cases of the stomach and duodenum decreased. Year by year, older patients have been increasing for procedures across all organs (Table 1).

**TABLE 1:** Annual changes of surgeries by sex, age group, and organ for the selected 115 gastrointestinal operative procedures in the training curriculum for board certified surgeons in gastroenterology

Organ	Year	No. surgeries	Percentage by sex		Percentage according to age group (y)					
			Male	Female	<60	60 to <65	65 to <70	70 to <75	75 to <80	≥80
Esophagus	2011	7246	81.8	18.2	22.5	19.6	21.1	18.7	12.0	6.0
	2012	8819	82.2	17.8	22.1	19.7	20.0	19.5	12.9	6.0
	2013	8642	81.5	18.5	20.8	17.5	21.0	20.6	13.2	6.9
	2014	9021	81.5	18.4	20.8	16.5	21.4	20.9	13.8	6.6
	2015	8943	80.8	19.2	19.6	15.3	22.4	22.5	13.1	7.1
	2016	9212	79.6	20.4	20.1	14.4	22.9	20.5	14.5	7.5
	2017	9359	80.0	20.0	19.3	13.4	24.4	19.4	15.5	8.0
	2018	9286	78.4	21.6	19.0	12.8	21.3	21.6	16.7	8.7
Stomach and duodenum	2011	66 740	68.0	32.0	20.1	14.4	14.0	17.1	16.4	18.0
	2012	76 186	68.3	31.7	18.9	14.4	14.5	17.1	16.4	18.6
	2013	75 583	67.9	32.1	18.6	13.1	15.5	17.2	16.9	18.7
	2014	74 920	67.6	32.4	17.9	12.1	16.0	17.8	16.7	19.5
	2015	73 877	67.8	32.2	17.4	11.1	17.1	17.8	16.6	19.9
	2016	72 234	67.8	32.2	17.0	10.2	18.1	17.1	16.6	21.0
	2017	68 287	67.2	32.8	16.3	9.9	17.5	17.3	17.2	21.8
	2018	65 152	66.9	33.1	16.0	9.0	16.4	18.0	17.5	23.2
Small intestine and colon	2011	151 143	56.7	43.3	37.4	10.9	10.5	12.1	12.2	16.9
	2012	184 810	56.7	43.3	36.4	10.7	10.7	12.2	12.5	17.4
	2013	198 677	56.9	43.1	35.6	10.1	11.3	12.7	12.4	17.8
	2014	206 857	56.9	43.1	34.7	9.4	12.0	13.1	12.4	18.4
	2015	214 453	57.1	42.9	34.0	8.9	12.9	13.1	12.3	18.7
	2016	218 228	57.3	42.7	33.7	8.4	13.6	12.5	12.4	19.3
	2017	235 359	56.7	43.3	32.7	8.0	13.2	12.7	12.9	20.5
	2018	236 496	56.9	43.1	32.2	7.7	12.6	13.4	13.2	21.1
Rectum and anus	2011	41 061	59.1	40.9	22.0	16.1	14.6	15.4	14.2	17.7
	2012	49 704	58.3	41.7	22.3	14.8	14.6	15.5	14.3	18.5
	2013	49 980	58.0	42.0	20.9	13.9	15.2	16.1	14.6	19.3
	2014	51 454	58.3	41.7	20.4	13.1	16.0	16.4	14.2	19.9
	2015	56 092	57.8	42.2	22.3	11.8	16.7	15.7	14.0	19.4
	2016	55 666	57.3	42.7	22.0	11.1	17.9	15.0	13.6	20.4
	2017	56 144	56.7	43.3	22.2	10.2	17.3	15.1	14.2	21.0
	2018	56 162	56.9	43.1	22.2	9.8	15.9	15.8	14.6	21.6
Liver	2011	22 855	67.3	32.7	22.2	16.5	16.3	18.7	17.2	9.2
	2012	26 288	66.3	33.7	22.1	15.7	16.7	18.0	17.4	10.2
	2013	25 814	66.1	33.9	21.3	14.6	17.6	18.7	17.3	10.5
	2014	26 518	66.3	33.7	21.5	13.7	18.1	19.8	16.6	10.3
	2015	26 378	65.7	34.3	20.8	12.8	18.9	19.4	16.5	11.5
	2016	27 212	66.4	33.6	20.3	11.5	20.5	18.6	17.0	12.1
	2017	27 397	65.8	34.2	20.1	11.0	20.2	18.8	17.2	12.7
	2018	26 531	66.5	33.5	19.6	10.3	18.8	19.6	17.8	13.8
Gall bladder	2011	103 183	54.5	45.4	34.3	14.0	12.2	13.8	12.8	13.0
	2012	122 513	55.2	44.8	32.9	13.8	12.4	13.9	13.2	13.8
	2013	129 162	55.3	44.7	32.6	12.9	13.0	14.2	13.2	14.0
	2014	131 182	55.6	44.4	32.1	11.8	13.9	14.5	13.2	14.5
	2015	133 126	55.6	44.4	32.0	11.2	15.0	14.1	13.0	14.8
	2016	137 360	55.4	44.6	32.6	10.6	15.5	13.1	12.9	15.3
	2017	138 267	55.6	44.4	32.2	10.2	15.1	13.5	13.2	15.8
	2018	139 844	55.3	44.7	31.8	9.7	14.2	14.2	13.4	16.7
Pancreas	2011	13 477	59.9	40.1	20.0	15.6	16.9	19.7	17.7	10.2
	2012	15 550	60.0	40.0	19.8	15.2	17.0	19.5	18.2	10.3
	2013	16 380	59.7	40.3	19.1	13.6	18.0	20.7	17.7	10.9
	2014	17 313	59.5	40.5	18.4	12.4	19.0	21.0	18.2	11.1
	2015	17 407	59.1	40.9	18.2	11.3	19.4	21.6	18.1	11.4
	2016	18 238	58.9	41.1	18.2	10.4	19.9	20.4	19.0	12.2
	2017	19 138	59.2	40.8	17.7	9.9	19.5	19.9	20.1	12.9
	2018	19 152	58.6	41.4	16.9	9.2	18.2	21.5	20.4	13.7
Spleen	2011	3609	61.3	38.7	35.3	15.6	14.7	14.8	11.9	7.8
	2012	4142	61.4	38.6	32.9	16.3	15.0	15.1	12.9	7.8
	2013	4509	61.8	38.2	30.8	14.9	15.9	16.5	13.1	8.7
	2014	4272	61.8	38.2	29.9	13.0	17.3	17.0	13.8	9.1
	2015	3568	60.4	39.6	29.7	11.4	17.3	16.6	14.1	10.8
	2016	3171	57.3	42.7	31.9	11.7	17.7	15.7	12.5	10.5
	2017	2864	58.7	41.3	31.6	11.0	18.1	16.0	13.3	10.0
	2018	2544	56.6	43.4	32.6	9.9	15.6	16.9	13.9	11.1
Others	2011	23 218	55.0	45.0	32.0	11.9	11.3	13.3	13.8	17.6
	2012	28 779	55.4	44.6	31.1	11.7	11.7	13.8	13.7	18.0
	2013	36 363	53.1	46.9	28.3	10.9	12.7	14.1	14.8	19.1
	2014	39 854	53.7	46.3	28.1	10.1	13.1	14.5	14.4	19.8
	2015	41 465	53.2	46.8	27.4	9.4	14.0	14.5	14.2	20.6
	2016	43 523	54.0	46.0	27.5	9.2	14.6	13.5	14.0	21.2
	2017	45 622	54.1	45.9	27.0	8.2	14.7	13.5	14.6	21.9
	2018	46 587	54.1	45.9	26.8	8.2	14.0	14.4	14.7	21.9

**FIGURE 1:**
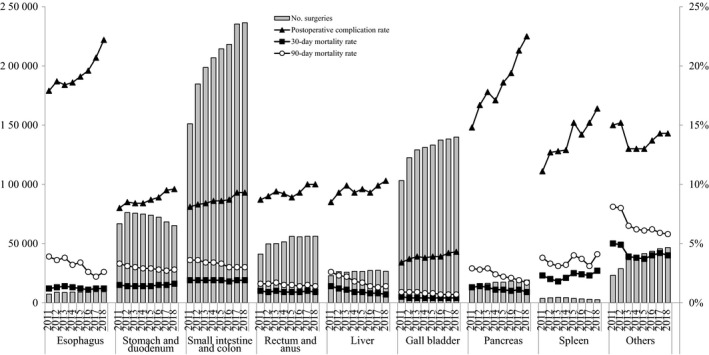
Annual changes of number of surgeries, 30‐Day mortality, operative mortality, and complications: Analysis of 115 surgical procedures. Postoperative complication rate: the rate of Clavien–Dindo (C–D) classification grade III (complications requiring intervention) or higher complications

In terms of the institutional groups in which the surgeries were performed, more than 70% of all surgeries were performed at certified institutions, and the percentage of surgeries performed at certified institutions has been increasing for all organs (Table 2). Also, more than 70% of the surgeries were performed with the participation of the board‐certified surgeon. The percentage of certified surgeons that were operators was high for the esophagus (75.2% in 2018), liver (64.1%), and pancreas (66.5%). The complication rates were comparatively higher and increasing for the esophagus and the pancreas, however, the mortality rates for procedures on these organs were not so high (Table 3 and Figure 1). Tables 4, 5, 6, 7, 8, 9, 10, 11, 12 show the number of surgeries, endoscopic surgeries, and morbidity/mortality rates according to the selected 115 gastrointestinal operative procedures in 2018.

**TABLE 2:** Institution and anesthesiologist and specialist participation rates by organ for the selected 115 gastrointestinal operative procedures

Organ	Year	No. surgeries	Percentage by institution group			Anesthesiologist Participation (%)	Board‐certified surgeon participation (%)	Medical practitioners (%)	
			Certified institution	Related institution	Other			Board‐certified Surgeons	Non‐board‐certified surgeons
Esophagus	2011	7246	93.5	5.9	0.6	97.0	87.0	62.8	37.2
	2012	8819	78.1	5.9	16.0	97.4	87.0	62.7	37.3
	2013	8642	90.6	7.1	2.4	97.3	88.4	64.4	35.6
	2014	9021	91.1	6.1	2.8	97.9	90.1	67.6	32.4
	2015	8943	91.5	6.0	2.5	97.9	91.1	69.4	30.6
	2016	9212	92.4	5.0	2.6	98.2	91.2	70.0	30.0
	2017	9359	92.7	4.0	3.3	97.9	92.5	71.8	28.2
	2018	9286	93.8	4.0	2.2	98.5	94.7	75.2	24.8
Stomach and duodenum	2011	66 740	80.2	17.3	2.6	92.8	69.3	35.1	64.9
	2012	76 186	63.5	15.6	20.9	93.5	70.3	35.6	64.4
	2013	75 583	76.3	19.3	4.4	93.3	73.5	37.7	62.3
	2014	74 920	77.0	18.2	4.8	93.6	75.9	39.2	60.8
	2015	73 877	77.1	18.3	4.6	93.9	76.1	39.2	60.8
	2016	72 234	79.6	16.1	4.3	94.6	78.7	41.0	59.0
	2017	68 287	79.6	15.3	5.1	94.8	79.7	41.8	58.2
	2018	65 152	80.0	14.8	5.1	95.1	81.4	43.2	56.8
Small intestine and colon	2011	151 143	76.8	20.2	2.9	88.1	59.2	25.1	74.9
	2012	184 810	60.6	18.2	21.2	88.9	59.9	25.4	74.6
	2013	198 677	72.6	22.2	5.2	89.6	62.7	26.6	73.4
	2014	206 857	73.0	21.4	5.6	90.8	65.4	28.1	71.9
	2015	214 453	73.8	20.7	5.5	91.6	66.3	28.5	71.5
	2016	218 228	75.6	19.0	5.5	92.4	68.1	29.5	70.5
	2017	235 359	76.0	18.0	6.0	92.9	70.1	31.1	68.9
	2018	236 496	76.3	17.5	6.1	93.3	71.8	32.6	67.4
Rectum and anus	2011	41 061	76.9	19.0	4.1	86.3	68.3	36.9	63.1
	2012	49 704	60.4	18.2	21.4	85.7	68.6	37.6	62.4
	2013	49 980	72.9	21.7	5.4	87.3	71.2	39.4	60.6
	2014	51 454	73.5	20.9	5.6	87.9	73.7	41.6	58.4
	2015	56 092	72.5	20.8	6.7	84.9	73.5	41.5	58.5
	2016	55 666	74.1	19.4	6.6	85.7	74.7	42.1	57.9
	2017	56 144	73.8	18.2	8.0	84.8	76.1	43.9	56.1
	2018	56 162	74.1	17.9	8.0	85.2	77.2	46.7	53.3
Liver	2011	22 855	89.3	9.7	1.1	95.6	85.2	55.2	44.8
	2012	26 288	74.2	9.2	16.7	95.4	85.7	57.4	42.6
	2013	25 814	86.3	10.7	2.9	96.3	87.5	57.1	42.9
	2014	26 518	86.3	10.0	3.7	96.4	89.0	59.6	40.4
	2015	26 378	87.3	9.5	3.2	96.6	89.1	59.1	40.9
	2016	27 212	88.4	8.8	2.9	96.8	90.0	59.6	40.4
	2017	27 397	89.0	7.8	3.1	97.1	91.8	62.5	37.5
	2018	26 531	89.4	7.1	3.5	97.3	92.8	64.1	35.9
Gall bladder	2011	103 183	73.9	22.5	3.6	91.8	61.9	26.4	73.6
	2012	122 513	57.5	19.6	22.9	92.1	62.8	26.3	73.7
	2013	129 162	69.9	24.1	5.9	92.2	65.4	27.3	72.7
	2014	131 182	70.3	23.3	6.4	92.3	67.4	28.1	71.9
	2015	133 126	70.8	22.8	6.4	92.9	68.4	28.1	71.9
	2016	137 360	72.4	21.3	6.3	93.5	69.4	28.9	71.1
	2017	138 267	72.6	20.1	7.3	93.7	71.4	29.9	70.1
	2018	139 844	72.5	20.1	7.4	94.1	73.1	31.1	68.9
Pancreas	2011	13 477	88.1	10.8	1.2	95.8	85.2	57.7	42.3
	2012	15 550	72.8	8.7	18.5	96.3	86.5	59.9	40.1
	2013	16 380	86.5	11.0	2.4	95.9	87.6	60.2	39.8
	2014	17 313	86.9	9.9	3.3	96.2	89.1	61.3	38.7
	2015	17 407	88.4	9.1	2.4	96.4	90.3	61.6	38.4
	2016	18 238	89.8	8.0	2.3	96.8	91.1	62.4	37.6
	2017	19 138	90.4	7.1	2.5	97.2	92.3	63.9	36.1
	2018	19 152	91.3	6.4	2.3	97.3	93.4	66.5	33.5
Spleen	2011	3609	87.0	11.6	1.4	94.6	75.2	44.9	55.1
	2012	4142	70.5	9.5	20.0	81.7	75.8	44.4	55.6
	2013	4509	83.2	13.8	3.0	95.2	75.4	43.3	56.7
	2014	4272	85.4	11.5	3.1	94.6	77.5	45.2	54.8
	2015	3568	85.6	12.3	2.1	94.8	78.9	45.5	54.5
	2016	3171	86.8	10.1	3.1	95.7	80.5	48.0	52.0
	2017	2864	87.4	9.3	3.3	95.3	82.3	49.1	50.9
	2018	2544	86.9	9.7	3.4	95.3	84.7	49.3	50.7
Others	2011	23 218	80.2	17.0	2.8	90.3	60.4	27.2	72.8
	2012	28 779	65.7	15.2	19.1	91.0	61.1	27.6	72.4
	2013	36 363	76.1	19.3	4.6	91.5	63.4	28.5	71.5
	2014	39 854	76.6	18.2	5.1	91.9	64.9	29.7	70.3
	2015	41 465	78.0	17.2	4.8	92.4	65.6	29.4	70.6
	2016	43 523	79.4	15.8	4.8	92.7	67.3	30.3	69.7
	2017	45 622	80.1	14.8	5.1	93.1	69.7	32.3	67.7
	2018	46 587	80.2	14.2	5.7	93.8	71.2	33.1	66.9

**TABLE 3:** Number of surgeries and mortality rates according to organ treated using the selected 115 gastrointestinal operative procedures

Organ	Year	No. surgeries	Number of postoperative complications^a^/rate (%)	Number of postoperative 30‐d mortalities/rate (%)	Number of postoperative 90‐d mortalities/rate (%)
Esophagus	2011	7246	1294/17.9	87/1.2	279/3.9
	2012	8819	1653/18.7	117/1.3	315/3.6
	2013	8642	1593/18.4	121/1.4	327/3.8
	2014	9021	1679/18.6	115/1.3	289/3.2
	2015	8943	1709/19.1	103/1.2	304/3.4
	2016	9212	1805/19.6	100/1.1	238/2.6
	2017	9359	1938/20.7	108/1.2	208/2.2
	2018	9286	2065/22.2	108/1.2	246/2.6
Stomach and duodenum	2011	66 740	5354/8.0	992/1.5	2183/3.3
	2012	76 186	6447/8.5	1085/1.4	2381/3.1
	2013	75 583	6380/8.4	1059/1.4	2269/3.0
	2014	74 920	6328/8.4	1064/1.4	2174/2.9
	2015	73 877	6418/8.7	1007/1.4	2110/2.9
	2016	72 234	6413/8.9	1066/1.5	2016/2.8
	2017	68 287	6455/9.5	1046/1.5	1863/2.7
	2018	65 152	6228/9.6	1048/1.6	1833/2.8
	2011	151 143	12 184/8.1	2943/1.9	5390/3.6
	2012	184 810	15 395/8.3	3564/1.9	6583/3.6
	2013	198 677	16 709/8.4	3723/1.9	6803/3.4
	2014	206 857	17 776/8.6	3822/1.9	6961/3.4
	2015	214 453	18 372/8.6	4019/1.9	7092/3.3
	2016	218 228	19 020/8.7	3933/1.8	6621/3.0
	2017	235 359	21 854/9.3	4588/1.9	7118/3.0
	2018	236 496	21 881/9.3	4452/1.9	7116/3.0
Rectum and anus	2011	41 061	3584/8.7	395/1.0	676/1.6
	2012	49 704	4488/9.0	462/0.9	802/1.6
	2013	49 980	4684/9.4	517/1.0	858/1.7
	2014	51 454	4711/9.2	449/0.9	792/1.5
	2015	56 092	4986/8.9	519/0.9	824/1.5
	2016	55 666	5194/9.3	503/0.9	766/1.4
	2017	56 144	5600/10.0	556/1.0	829/1.5
	2018	56 162	5622/10.0	522/0.9	803/1.4
Liver	2011	22 855	1933/8.5	309/1.4	590/2.6
	2012	26 288	2454/9.3	310/1.2	605/2.3
	2013	25 814	2549/9.9	275/1.1	575/2.2
	2014	26 518	2466/9.3	246/0.9	481/1.8
	2015	26 378	2537/9.6	234/0.9	451/1.7
	2016	27 212	2543/9.3	222/0.8	382/1.4
	2017	27 397	2724/9.9	214/0.8	364/1.3
	2018	26 531	2737/10.3	189/0.7	372/1.4
Gall bladder	2011	103 183	3473/3.4	483/0.5	946/0.9
	2012	122 513	4587/3.7	531/0.4	1082/0.9
	2013	129 162	4982/3.9	546/0.4	1130/0.9
	2014	131 182	5020/3.8	569/0.4	1097/0.8
	2015	133 126	5231/3.9	541/0.4	1036/0.8
	2016	137 360	5320/3.9	559/0.4	980/0.7
	2017	138 267	5761/4.2	576/0.4	968/0.7
	2018	139 844	5964/4.3	584/0.4	954/0.7
Pancreas	2011	13 477	1994/14.8	175/1.3	386/2.9
	2012	15 550	2595/16.7	213/1.4	437/2.8
	2013	16 380	2917/17.8	211/1.3	482/2.9
	2014	17 313	2966/17.1	195/1.1	423/2.4
	2015	17 407	3229/18.6	185/1.1	379/2.2
	2016	18 238	3543/19.4	185/1.0	390/2.1
	2017	19 138	4076/21.3	219/1.1	365/1.9
	2018	19 152	4309/22.5	178/0.9	325/1.7
Spleen	2011	3609	400/11.1	83/2.3	137/3.8
	2012	4142	528/12.7	84/2.0	138/3.3
	2013	4509	575/12.8	79/1.8	139/3.1
	2014	4272	549/12.9	88/2.1	137/3.2
	2015	3568	543/15.2	88/2.5	144/4.0
	2016	3171	449/14.2	76/2.4	117/3.7
	2017	2864	434/15.2	65/2.3	89/3.1
	2018	2544	418/16.4	69/2.7	104/4.1
Others	2011	23 218	3494/15.0	1163/5.0	1887/8.1
	2012	28 779	4388/15.2	1399/4.9	2293/8.0
	2013	36 363	4712/13.0	1401/3.9	2346/6.5
	2014	39 854	5176/13.0	1521/3.8	2489/6.2
	2015	41 465	5380/13.0	1541/3.7	2545/6.1
	2016	43 523	5975/13.7	1760/4.0	2684/6.2
	2017	45 622	6539/14.3	1909/4.2	2699/5.9
	2018	46 587	6645/14.3	1865/4.0	2710/5.8

^a^ Complications were defined by Clavien‐Dindo gradeⅢa‐Ⅴ.

**TABLE 4:** Number of surgeries, endoscopic surgery, and mortality rates according to the selected 115 gastrointestinal operative procedures in 2018 (esophagus)

Organ	Degree of difficulty	Procedure	No. surgeries	No. Endoscopic surgeries/ rate (%)	No. postoperative complications*/rate (%)	No. postoperative 30‐d mortalities /rate (%)	No. postoperative 30‐d mortalities/rate (%)
Esophagus	Low	Cervical periesophageal abscess drainage	40	5/12.5	13/32.5	2/5.0	2/5.0
	Med	Esophageal suture (perforation, injury)	211	22/10.4	81/38.4	9/4.3	12/5.7
	Med	Thoracic periesophageal abscess drainage	27	1/3.7	15/55.6	1/3.7	3/11.1
	Med	Esophageal foreign body extraction	36	2/5.6	9/25.0	1/2.8	2/5.6
	Med	Esophageal diverticulum resection	53	26/49.1	2/3.8	0/0.0	0/0
	Med	Benign esophageal tumor removal	76	48/63.2	5/6.6	0/0	1/1.3
	Med	Esophageal resection (removal only)	687	403/58.7	102/14.8	12/1.7	31/4.5
	Med	Esophageal reconstruction: reconstruction only (gastric tube reconstruction)	647	372/57.5	157/24.3	7/1.1	15/2.3
	Med	Esophageal fistula construction	192	69/35.9	56/29.2	11/5.7	22/11.5
	Med	Esophagocardioplasty	306	210/68.6	19/6.2	2/0.7	2/0.7
	Med	Achalasia surgery	232	162/69.8	2/0.9	0/0	0/0
	High	Esophagectomy	6207	3788/61.0	1420/22.9	53/0.9	115/1.9
	High	Esophageal reconstruction: reconstruction only (colon reconstruction)	31	15/48.4	10/32.3	0/0	2/6.5
	High	Esophageal bypass	152	18/11.8	55/36.2	6/3.9	18/11.8
	High	Bronchoesophageal fistula surgery	3	1/33.3	2/66.7	0/0	0/0
	High	Secondary esophageal reconstruction	386	39/10.1	117/30.3	4/1.0	21/5.4

**TABLE 5:** Number of surgeries, endoscopic surgery, and mortality rates according to the selected 115 gastrointestinal operative procedures in 2018 (stomach and duodenum）

Organ	Degree of difficulty	Procedure	No. surgeries	No. Endoscopic surgeries/ rate (%)	No. postoperative complications*/rate (%)	No. postoperative 30‐d mortalities/rate (%)	No. postoperative 90‐d mortalities/rate (%)
Stomach and duodenum	Low	Gastrostomy and suture gastrorrhaphy	61	9/14.8	3/4.9	3/4.9	3/4.9
	Low	Diverticulum, polypectomy (excluding endoscopic resection)	169	19/11.2	19/11.2	2/1.2	3/1.8
	Low	Truncal vagotomy	4	2/50.0	1/25.0	1/25.0	1/25.0
	Low	Gastroenterostomy (Including duodenal jejunostomy)	5882	1406/23.9	974/16.6	300/5.1	615/10.5
	Low	Gastric fistula construction (Excluding PEG)	1560	244/15.6	303/19.4	98/6.3	162/10.4
	Low	Gastric pyloroplasty	66	12/18.2	8/12.1	1/1.5	2/3.0
	Low	Gastric volvulus (volvulus) surgery and rectopexy	53	29/54.7	4/7.5	0/0	0/0
	Low	Gastric suture (including gastric suture for gastric rupture, Suture closure for gastroduodenal perforation, omental implantation and omental transposition）	5801	1975/34.0	925/15.9	250/4.3	361/6.2
	Low	Local gastrectomy (including wedge resection)）	4338	2925/67.4	135/3.1	18/0.4	28/0.6
	Med	Gastrectomy (including distal gastrectomy, pylorus preserving gastrectomy and segmental [transverse] gastrectomy)	33 988	16 355/48.1	2327/6.8	227/0.7	393/1.2
	Med	Selective vagotomy	7	2/28.6	0/0	0/0	0/0
	High	Total gastrectomy (including fundusectomy)）	13 223	3344/25.3	1529/11.6	148/1.1	265/2.0
	High	Left upper abdominal exenteration	0	0/0	0/0	0/0	0/0

**TABLE 6:** Number of surgeries, endoscopic surgery, and mortality rates according to the selected 115 gastrointestinal operative procedures in 2018 (small intestine and colon)

Organ	Degree of difficulty	Procedure	No. surgeries	No. Endoscopic surgeries/ rate (%)	No. postoperative complications*/rate (%)	No. postoperative 30‐d mortalities/rate (%)	No. postoperative 90‐d mortalities/rate (%)
Small intestine and colon	Low	Enterotomy and enterorrhaphy	4501	684/15.2	791/17.6	183/4.1	392/8.7
	Low	Disinvagination (invasive)	225	62/27.6	20/8.9	12/5.3	13/5.8
	Low	Partial enterectomy (benign)	9381	1601/17.1	1845/19.7	572/6.1	830/8.8
	Low	Ileocecal resection (benign)	4784	1765/36.9	452/9.4	83/1.7	118/2.5
	Low	Partial colectomy and sigmoid colectomy (benign)	8073	2319/28.7	1243/15.4	301/3.7	430/5.3
	Low	Appendectomy	54 877	35 558/64.8	1001/1.8	58/0.1	89/0.2
	Low	Enterostomy and closure (without enterectomy)	26 086	8163/31.3	4482/17.2	1013/3.9	1796/6.9
	Med	Enterectomy (malignant)	3717	927/24.9	446/12.0	85/2.3	157/4.2
	Med	Ileocecal resection (malignant)	13 858	7995/57.7	756/5.5	87/0.6	162/1.2
	Med	Partial colectomy and sigmoid colectomy (malignant)	32 212	18 626/57.8	2176/6.8	199/0.6	336/1.0
	Med	Right hemicolectomy	22 610	11 165/49.4	1679/7.4	276/1.2	424/1.9
	Med	Left hemicolectomy	6072	2964/48.8	667/11.0	124/2.0	177/2.9
	Med	Total colectomy	1760	489/27.8	404/23.0	144/8.2	179/10.2
	Med	Intestinal obstruction surgery (with bowel resection)	24 572	5248/21.4	2532/10.3	577/2.3	835/3.4
	Med	Enterostomy and closure (with enterectomy)	23 313	3722/16.0	3325/14.3	734/3.1	1173/5.0
	High	Proctocolectomy and ileoanal (canal) anastomosis	455	195/42.9	62/13.6	4/0.9	5/1.1

**TABLE 7:** Number of surgeries, endoscopic surgery, and mortality rates according to the selected 115 gastrointestinal operative procedures in 2018 (rectum and anus)

Organ	Degree of difficulty	Procedure	No. surgeries	No. Endoscopic surgeries/ rate (%)	No. postoperative complications*/rate (%)	No. postoperative 30‐d mortalities/rate (%)	No. postoperative 90‐d mortalities/rate (%)
Rectum	Low	Transanal rectal tumor removal	3751	103/2.7	22/0.6	2/0.1	3/0.1
	Low	Proctocele surgery (transanal)	2870	29/1.0	49/1.7	9/0.3	14/0.5
	Med	Rectectomy (benign)	1371	118/8.6	265/19.3	23/1.7	43/3.1
	Med	High anterior resection	10 741	7081/65.9	744/6.9	50/0.5	78/0.7
	Med	Hartmann's procedure	6075	1040/17.1	1238/20.4	309/5.1	448/7.4
	Med	Proctocele surgery (abdominoperineal)	1993	1058/53.1	31/1.6	9/0.5	10/0.5
	Med	Malignant anorectal tumor excision (transanal)	663	80/12.1	15/2.3	1/0.2	1/0.2
	Med	Anal sphincteroplasty (by tissue replacement)	2641	15/0.6	16/0.6	4/0.2	6/0.2
	High	Rectectomy (malignant)	4935	3144/63.7	660/13.4	23/0.5	49/1.0
	High	Low anterior resection	20 636	13 825/67.0	2454/11.9	90/0.4	142/0.7
	High	Pelvic evisceration	441	85/19.3	121/27.4	2/0.5	9/2.0
	High	Anorectal malignant tumor excision (posterior approach)	45	5/11.1	7/15.6	0/0	0/0

**TABLE 8:** Number of surgeries, endoscopic surgery, and mortality rates according to the selected 115 gastrointestinal operative procedures in 2018 (liver)

Organ	Degree of difficulty	Procedure	No. surgeries	No. Endoscopic surgeries/ rate (%)	No. postoperative complications*/rate (%)	No. postoperative 30‐day mortalities/rate (%)	No. postoperative 90‐day mortalities/rate (%)
Liver	Low	Hepatorrhaphy	54	3/5.6	21/38.9	8/14.8	9/16.7
	Low	Liver abscess drainage (excluding percutaneous procedures)	47	4/8.5	9/19.1	0/0.0	1/2.1
	Low	Hepatic cyst resection. Suture. Drainage	794	618/77.8	42/5.3	3/0.4	5/0.6
	Low	Partial hepatectomy	12 543	4072/32.5	940/7.5	52/0.4	96/0.8
	Low	Liver biopsy (excluding percutaneous procedures)	235	41/17.4	10/4.3	4/1.7	7/3.0
	Low	Liver coagulonecrotic therapy (excluding percutaneous procedures)	705	143/20.3	57/8.1	6/0.9	15/2.1
	Med	Lateral segmentectomy of the liver	1626	564/34.7	90/5.5	5/0.3	14/0.9
	Med	Esophageal and gastric varix surgery	42	29/69.0	5/11.9	1/2.4	2/4.8
	High	Hepatectomy (segmented or more; excluding lateral segments)	7192	791/11.0	1137/15.8	83/1.2	163/2.3
	High	Systematic subsegmentectomy	2474	455/18.4	210/8.5	7/0.3	23/0.9
	High	Liver transplant	705	1/0.1	165/23.4	17/2.4	33/4.7
	High	Hepatopancreatoduodenectomy	114	2/1.8	51/44.7	3/2.6	4/3.5

**TABLE 9:** Number of surgeries, endoscopic surgery, and mortality rates according to the selected 115 gastrointestinal operative procedures in 2018 (gall bladder)

Organ	Degree of difficulty	Procedure	No. surgeries	No. Endoscopic surgeries/ rate (%)	No. postoperative complications*/rate (%)	No. postoperative 30‐d mortalities/rate (%)	No. postoperative 90‐d mortalities/rate (%)
Gall bladder	Low	Cholangiotomy	100	10/10.0	16/16.0	1/1.0	1/1.0
	Low	Cysticolithectomy	60	8/13.3	12/20.0	1/1.7	1/1.7
	Low	Cholecystectomy	132 548	90 831/68.5	4782/3.6	469/0.4	735/0.6
	Low	External cholecystostomy	121	16/13.2	27/22.3	10/8.3	15/12.4
	Low	Cystoenteric anastomosis	53	8/15.1	12/22.6	2/3.8	5/9.4
	Med	Cysticolithectomy	2664	693/26.0	248/9.3	25/0.9	55/2.1
	Med	Biliary tract reconstruction	336	13/3.9	75/22.3	2/0.6	3/0.9
	Med	Biliary bypass	1210	67/5.5	187/15.5	31/2.6	62/5.1
	Med	Cholangioplasty	113	16/14.2	22/19.5	3/2.7	6/5.3
	Med	Duodenal papilloplasty	32	1/3.1	6/18.8	0/0	0/0
	Med	Choledocal dilatation	289	45/15.6	29/10.0	0/0.0	1/0.3
	Med	Biliary fistula closure	38	7/18.4	8/21.1	1/2.6	1/2.6
	High	Malignant gallbladder tumor surgery (excluding simple cholecystectomy)）	1007	39/3.9	117/11.6	6/0.6	8/0.8
	High	Malignant bile duct tumor surgery	1263	12/1.0	423/33.5	33/2.6	61/4.8
	High	Biliary atresia surgery	10	0/0	0/0	0/0	0/0

**TABLE 10:** Number of surgeries, endoscopic surgery, and mortality rates according to the selected 115 gastrointestinal operative procedures in 2018 (pancreas)

Organ	Degree of difficulty	Procedure	No. of surgeries	No. Endoscopic surgeries/ rate (%)	No. postoperative complications*/rate (%)	No. postoperative 30‐d mortalities/rate (%)	No. postoperative 90‐d mortalities/rate (%)
Pancreas	Low	External pancreatic cyst drainage	21	4/19.0	11/52.4	1/4.8	2/9.5
	Low	External pancreatic duct drainage	6	0/0.0	2/33.3	0/0.0	1/16.7
	Med	Pancreatorrhaphy	13	0/0.0	8/61.5	3/23.1	4/30.8
	Med	Partial pancreatic resection	162	36/22.2	36/22.2	0/0.0	1/0.6
	Med	Distal pancreatectomy (benign)	1493	599/40.1	293/19.6	5/0.3	9/0.6
	Med	Pancreatoenteric anastomosis	28	1/3.6	3/10.7	1/3.6	2/7.1
	Med	Pancreatic (duct) anastomosis	286	4/1.4	48/16.8	7/2.4	9/3.1
	Med	Acute pancreatitis surgery	88	8/9.1	41/46.6	5/5.7	12/13.6
	Med	Pancreatolithiasis surgery	8	0/0	1/12.5	0/0	0/0
	Med	Plexus pancreaticus capitalis resection	0	0/	0/	0/	0/
	High	Pancreaticoduodenectomy	11 626	194/1.7	2716/23.4	111/1.0	204/1.8
	High	Distal pancreatectomy (malignant)）	4611	795/17.2	971/21.1	19/0.4	42/0.9
	High	Total pancreatectomy	599	9/1.5	105/17.5	25/4.2	38/6.3
	High	Duodenum preserving pancreas head resection	49	0/0.0	14/28.6	0/0.0	0/0
	High	Segmental pancreatic resection	136	5/3.7	52/38.2	1/0.7	1/0.7
	High	Distal pancreatectomy	26	0/0	8/30.8	0/0	0/0

**TABLE 11:** Number of surgeries, endoscopic surgery, and mortality rates according to the selected 115 gastrointestinal operative procedures in 2018 (spleen)

Organ	Degree of difficulty	Procedure	No. surgeries	No. Endoscopic surgeries/ rate (%)	No. postoperative complications*/rate (%)	No. postoperative 30‐d mortalities/rate (%)	No. postoperative 90‐d mortalities/rate (%)
Spleen	Low	Splenorrhaphy	23	4/17.4	6/26.1	1/4.3	1/4.3
	Med	Splenectomy	2490	694/27.9	409/16.4	68/2.7	103/4.1
	Med	Partial splenic resection	31	16/51.6	3/9.7	0/0	0/0

**TABLE 12:** Number of surgeries, endoscopic surgery, and mortality rates according to the selected 115 gastrointestinal operative procedures in 2018 (other)

Organ	Degree of difficulty	Procedure	No. surgeries	No. Endoscopic surgeries/ rate (%)	No. postoperative complications*/rate (%)	No. postoperative 30‐d mortalities/rate (%)	No. postoperative 90‐d mortalities/rate (%)
Other	Low	Localized intra‐abdominal abscess surgery	2617	645/24.6	400/15.3	56/2.1	114/4.4
	Low	Exploratory laparotomy	10 825	5335/49.3	1329/12.3	515/4.8	718/6.6
	Med	Acute diffuse peritonitis surgery	14 835	2820/19.0	3943/26.6	1117/7.5	1617/10.9
	Med	Ventral hernia surgery	13 768	3931/28.6	497/3.6	84/0.6	117/0.8
	Med	Diaphragm suture	243	76/31.3	30/12.3	4/1.6	6/2.5
	Med	Esophageal hiatus hernia surgery	1033	668/64.7	81/7.8	19/1.8	25/2.4
	Med	Retroperitoneal tumor surgery	866	129/14.9	60/6.9	5/0.6	10/1.2
	Med	Abdominal wall/mesenteric/omental tumor resection	1832	422/23.0	151/8.2	25/1.4	41/2.2
	Med	Gastrointestinal perforation closure	511	60/11.7	149/29.2	39/7.6	59/11.5
	High	Diaphragmatic hiatus hernia surgery	57	17/29.8	5/8.8	1/1.8	3/5.3

### 3.2 Eight main operative procedures

**FIGURE 2:**
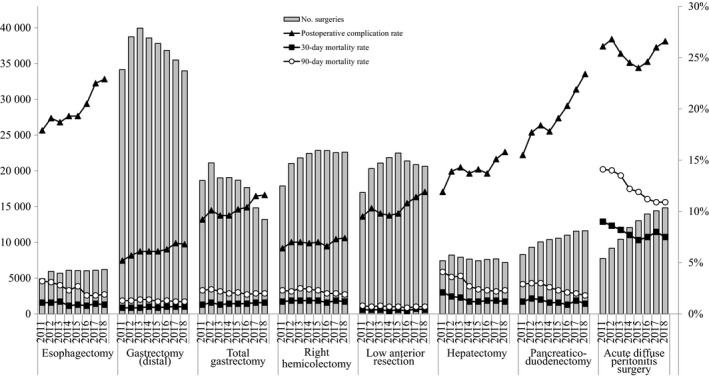
Annual changes of number of surgeries, 30‐Day mortality, operative mortality, and complications: Analysis of 8 major surgical procedures. Postoperative complication rate: the rate of C–D classification grade III or higher complications

The number of surgeries carried out annually for the eight main operative procedures, the percentage by gender, and the percentage according to age group between 2011 and 2018 are shown in Table 13. The percentages of elderly patients have been increasing, especially for gastrectomy (distal and total), right hemicolectomy, and acute diffuse peritonitis surgery. Regarding the Institutional groups in which surgeries were performed, more than 70% of the surgeries were performed at certified institutions, and more than 90% were for esophagectomy, hepatectomy (non‐lateral segments), and pancreaticoduodenectomy in 2018 (Table 14). Board‐certified surgeon participation rates have been increasing year by year for all procedures. Table 15 shows the morbidity and mortality rates of the eight main operative procedures. Other than acute diffuse peritonitis surgery, the postoperative 30‐day mortality rate was 0.4%‐1.2% and the operative mortality rate was 0.7%‐2.3%; a very low level relatively. The postoperative 30‐day mortality rate and operative mortality rate for acute diffuse peritonitis surgery was 7.5% and 10.9% in 2018, respectively. Figure 2 shows the annual changes in the number of surgeries, postoperative complication rate, and mortalities. Although the complication rates were increasing for esophagectomy (22.9% in 2018) and pancreaticoduodenectomy (23.4%), the mortality rates for these procedures (0.9% and 1.9%, 1.0% and 1.8%, in 2018, respectively) were decreasing. The case numbers for acute diffuse peritonitis surgery were increasing; however, the morbidity and mortality rates were decreasing.

**TABLE 13:** Annual changes of surgeries by sex, age group, and organ for eight main operative procedures

Procedure	Year	No. surgeries	Percentage by sex		Percentage according to age group (y)					
			Male	Female	<60	60 to < 65	65 to < 70	70 to < 75	75 to < 80	≥80
Esophagectomy	2011	4916	84.1	15.9	20.4	20.8	22.5	19.4	12.2	4.7
	2012	5946	84.4	15.6	19.7	21.3	20.7	20.3	13.1	4.9
	2013	5694	83.6	16.4	18.3	18.3	22.6	21.3	13.8	5.8
	2014	6091	84.0	16.0	18.7	17.8	22.8	22.0	13.4	5.2
	2015	6060	82.9	17.1	17.9	16.3	23.6	23.5	13.1	5.7
	2016	6041	81.7	18.3	17.8	15.8	25.3	21.6	14.3	5.2
	2017	6100	82.3	17.7	17.0	14.6	25.6	20.6	15.8	6.3
	2018	6207	80.5	19.5	17.2	14.2	22.6	22.8	16.8	6.5
Gastrectomy (distal)	2011	34 160	66.6	33.4	18.1	15.0	14.2	17.4	16.8	18.5
	2012	38 750	66.9	33.1	16.9	14.8	15.0	17.8	16.5	18.8
	2013	39 957	66.7	33.3	16.3	13.5	15.8	17.8	17.6	19.0
	2014	38 584	66.4	33.6	15.7	12.4	16.6	18.4	17.3	19.5
	2015	37 819	66.6	33.4	14.8	11.3	17.5	18.2	17.5	20.6
	2016	36 852	66.6	33.4	14.5	10.4	18.5	17.6	17.4	21.6
	2017	35 517	66.8	33.2	13.4	9.9	18.0	18.1	18.0	22.6
	2018	33 988	66.6	33.4	12.9	9.1	16.9	19.0	18.1	24.0
Total gastrectomy	2011	18 652	73.7	26.3	16.6	14.7	16.0	19.7	18.0	15.0
	2012	21 122	74.2	25.8	15.5	14.8	15.7	19.2	18.5	16.3
	2013	19 035	74.0	26.0	14.7	13.5	16.9	19.4	19.2	16.3
	2014	19 071	73.7	26.3	14.0	12.3	17.2	20.1	18.9	17.5
	2015	18 695	74.5	25.5	13.7	11.1	18.9	20.8	18.2	17.4
	2016	17 670	74.4	25.6	12.6	10.3	19.6	19.5	19.0	19.0
	2017	14 840	74.2	25.8	12.2	9.9	19.0	19.6	19.8	19.5
	2018	13 223	74.4	25.6	10.8	9.1	18.0	20.6	20.6	20.9
Right hemicolectomy	2011	17 890	50.5	49.5	12.8	11.6	13.1	17.3	18.8	26.5
	2012	21 034	50.3	49.7	13.1	10.9	13.1	17.0	19.0	26.9
	2013	21 814	50.6	49.4	13.0	10.0	13.4	17.6	18.9	27.1
	2014	22 446	50.6	49.4	12.0	9.2	13.8	18.2	18.6	28.2
	2015	22 850	50.5	49.5	11.5	8.6	14.6	18.1	18.1	29.1
	2016	22 829	51.3	48.7	11.4	7.7	15.9	16.7	18.5	29.8
	2017	22 543	50.9	49.1	11.3	7.4	14.9	16.3	19.3	30.8
	2018	22 610	51.4	48.6	10.7	6.9	13.9	17.7	19.6	31.2
Low anterior resection	2011	16 984	64.8	35.2	24.1	18.5	16.5	16.2	12.9	11.7
	2012	20 321	64.8	35.2	24.2	17.6	16.5	16.6	13.1	12.0
	2013	21 096	64.2	35.8	23.8	16.5	17.4	16.9	13.5	11.8
	2014	21 861	64.8	35.2	23.1	15.7	18.3	17.9	13.1	11.9
	2015	22 493	64.4	35.6	23.5	14.2	19.6	17.1	13.6	12.0
	2016	21 387	64.4	35.6	23.4	13.6	20.7	16.8	13.2	12.2
	2017	20 879	64.2	35.8	23.2	12.6	20.9	16.7	13.5	13.2
	2018	20 636	64.9	35.1	22.9	12.5	19.3	18.0	14.4	12.9
Hepatectomy (non‐lateral segments)	2011	7434	70.4	29.6	20.1	16.4	16.5	20.4	18.0	8.7
	2012	8239	69.5	30.5	19.8	16.1	17.4	19.5	18.5	8.8
	2013	7937	69.4	30.6	19.4	14.2	18.0	20.3	18.2	9.9
	2014	7666	69.2	30.8	18.5	13.8	18.5	21.5	17.6	10.0
	2015	7439	68.9	31.1	18.7	12.5	19.3	20.9	17.6	11.1
	2016	7610	68.7	31.3	18.0	11.9	21.1	20.4	17.5	11.1
	2017	7698	69.5	30.5	17.2	11.3	20.5	20.4	18.7	11.9
	2018	7192	69.5	30.5	17.2	9.6	19.1	21.4	19.4	13.3
Pancreaticoduodenectomy	2011	8305	61.9	38.1	16.1	16.0	17.3	20.9	18.8	10.9
	2012	9329	62.0	38.0	14.7	15.8	18.0	20.6	20.2	10.6
	2013	10 068	60.9	39.1	14.0	12.6	19.6	22.5	19.4	11.8
	2014	10 400	59.5	40.5	18.4	12.4	19.0	21.0	18.2	11.1
	2015	10 576	60.7	39.3	14.2	11.7	20.0	22.9	19.3	12.0
	2016	11 028	61.1	38.9	14.2	10.3	20.6	21.8	20.3	12.7
	2017	11 580	61.1	38.9	13.8	9.8	20.4	20.8	21.6	13.6
	2018	11 626	60.3	39.7	13.3	9.1	18.9	22.2	22.0	14.6
Acute diffuse peritonitis surgery	2011	7753	60.0	40.0	31.4	11.2	9.7	11.7	13.2	22.9
	2012	9177	61.0	39.0	30.3	11.2	10.1	11.6	13.4	23.4
	2013	10 447	60.1	39.9	29.1	10.3	11.5	11.8	13.1	24.1
	2014	12 085	61.2	38.8	28.4	9.5	12.2	12.3	12.9	24.7
	2015	13 030	59.4	40.6	28.2	8.9	12.5	13.1	12.3	25.0
	2016	13 981	60.2	39.8	27.4	8.6	13.4	12.4	12.3	26.0
	2017	14 423	59.4	40.6	26.5	7.8	13.0	12.0	13.6	27.1
	2018	14 835	59.2	40.8	26.1	7.7	12.7	13.1	13.5	26.9

**TABLE 14:** Institution and anesthesiologist and specialist participation rates by organ for eight main operative procedures

Procedure	Year	No. surgeries	Percentage by institution group			Anesthesiologist Participation (%)	Board‐certified Surgeon participation (%)	Medical practitioners (%)	
			Certified institution	Related institution	Other			Board‐certified Surgeons	Non‐board‐certified surgeons
Esophagectomy	2011	4916	94.2	5.3	0.5	97.6	88.4	63.5	36.5
	2012	5946	78.3	4.9	16.8	98.1	89.0	64.8	35.2
	2013	5694	92.9	5.9	1.2	98.0	90.8	66.6	33.4
	2014	6091	93.6	4.7	1.7	98.6	92.6	70.2	29.8
	2015	6060	93.6	4.6	1.8	98.5	93.5	72.1	27.9
	2016	6041	94.5	3.8	1.7	98.8	93.7	73.2	26.8
	2017	6100	95.3	3.1	1.7	98.8	94.8	74.7	25.3
	2018	6207	95.9	2.7	1.4	99.1	96.6	78.8	21.2
Gastrectomy (distal)	2011	34 160	81.1	16.6	2.3	93.2	71.3	37.0	63.0
	2012	38 750	64.5	15.2	20.3	93.9	72.5	37.9	62.1
	2013	39 957	76.6	19.2	4.1	93.6	76.1	40.6	59.4
	2014	38 584	77.7	17.8	4.5	94.0	78.4	42.1	57.9
	2015	37 819	77.3	18.3	4.4	94.1	78.1	41.3	58.7
	2016	36 852	80.2	15.9	4.0	95.0	81.8	43.8	56.2
	2017	35 517	80.2	14.9	4.8	95.4	82.4	45.2	54.8
	2018	33 988	80.7	14.4	4.8	95.6	84.2	46.6	53.4
Total gastrectomy	2011	18 652	80.9	16.8	2.3	93.9	71.6	37.4	62.6
	2012	21 122	63.0	15.3	21.7	94.3	72.1	38.0	62.0
	2013	19 035	77.2	18.9	3.9	94.2	75.0	39.5	60.5
	2014	19 071	77.8	17.9	4.3	94.4	77.7	41.7	58.3
	2015	18 695	77.9	17.9	4.1	94.5	78.2	42.6	57.4
	2016	17 670	80.0	15.9	4.0	95.0	81.4	45.0	55.0
	2017	14 840	79.3	15.8	4.9	95.0	80.7	44.3	55.7
	2018	13 223	79.6	15.5	4.9	95.4	82.6	46.2	53.8
Right hemicolectomy	2011	17 890	75.7	21.2	3.1	92.7	66.0	30.5	69.5
	2012	21 034	60.0	18.3	21.7	93.0	67.1	30.8	69.2
	2013	21 814	72.1	22.3	5.6	92.9	69.7	32.6	67.4
	2014	22 446	71.2	23.1	5.7	93.4	71.9	33.6	66.4
	2015	22 850	72.1	22.0	5.9	94.1	72.4	33.5	66.5
	2016	22 829	73.8	20.1	6.1	94.5	74.2	34.3	65.7
	2017	22 543	75.0	18.4	6.6	94.7	76.4	37.1	62.9
	2018	22 610	74.8	19.0	6.2	94.7	77.8	38.2	61.8
Low anterior resection	2011	16 984	79.4	17.7	2.9	93.4	72.7	41.6	58.4
	2012	20 321	64.0	16.2	19.7	93.8	73.0	42.3	57.7
	2013	21 096	76.3	19.5	4.2	93.7	75.5	44.3	55.7
	2014	21 861	76.2	19.0	4.9	94.4	78.2	47.2	52.8
	2015	22 493	76.9	18.3	4.8	94.6	79.2	47.7	52.3
	2016	21 387	79.0	16.4	4.7	95.0	81.0	48.8	51.2
	2017	20 879	79.3	15.6	5.1	95.2	83.1	51.2	48.8
	2018	20 636	80.9	14.3	4.8	95.2	84.5	54.4	45.6
Hepatectomy (non‐lateral segments)	2011	7434	91.1	8.0	0.8	96.4	88.9	61.5	38.5
	2012	8239	75.9	7.9	16.3	96.8	89.3	64.0	36.0
	2013	7937	88.1	9.7	2.2	96.9	91.0	65.2	34.8
	2014	7666	88.2	8.7	3.1	96.7	92.3	66.6	33.4
	2015	7439	89.2	8.6	2.2	97.2	92.3	66.6	33.4
	2016	7610	90.7	7.1	2.1	97.1	93.3	67.7	32.3
	2017	7698	91.2	6.6	2.2	97.7	95.1	72.3	27.7
	2018	7192	92.8	5.2	2.0	97.7	95.8	72.8	27.2
Pancreaticoduodenectomy	2011	8305	87.8	11.0	1.2	95.9	85.7	58.7	41.3
	2012	9329	72.4	8.8	18.8	96.6	87.2	60.9	39.1
	2013	10 068	85.9	11.7	2.4	96.0	87.9	60.5	39.5
	2014	10 400	86.4	10.4	3.3	96.4	90.3	62.2	37.8
	2015	10 576	88.5	9.2	2.4	96.9	90.9	62.1	37.9
	2016	11 028	89.4	8.3	2.3	97.1	91.7	63.3	36.7
	2017	11 580	90.5	7.2	2.3	97.3	93.0	65.0	35.0
	2018	11 626	91.4	6.4	2.2	97.4	94.0	67.6	32.4
Acute diffuse peritonitis surgery	2011	7753	80.6	16.9	2.4	90.0	58.5	23.5	76.5
	2012	9177	65.2	16.4	18.4	90.4	59.4	22.7	77.3
	2013	10 447	77.7	18.1	4.2	91.2	62.4	23.9	76.1
	2014	12 085	77.7	17.2	5.1	91.9	63.3	25.1	74.9
	2015	13 030	79.8	15.9	4.3	92.2	64.5	24.9	75.1
	2016	13 981	82.2	13.8	4.0	93.0	66.8	26.1	73.9
	2017	14 423	83.1	13.0	3.8	93.3	69.0	27.2	72.8
	2018	14 835	83.4	12.4	4.2	93.6	70.4	28.7	71.3

**TABLE 15:** Number of surgeries and mortality rates according to organ treated using the eight main operative procedures

Procedure	Year	No. surgeries	No. postoperative complications*/rate (%)	No. re‐operation/rate (%)	No. postoperative 30‐d mortalities/rate (%)	No. postoperative 90‐d mortalities/rate (%)
Esophagectomy	2011	4916	879/17.9	310/6.3	55/1.1	158/3.2
	2012	5946	1135/19.1	345/5.8	63/1.1	183/3.1
	2013	5694	1067/18.7	375/6.6	67/1.2	161/2.8
	2014	6091	1178/19.3	367/6.0	49/0.8	140/2.3
	2015	6060	1171/19.3	392/6.5	57/0.9	166/2.7
	2016	6041	1240/20.5	357/5.9	49/0.8	109/1.8
	2017	6100	1374/22.5	355/5.8	61/1.0	108/1.8
	2018	6207	1420/22.9	367/5.9	53/0.9	115/1.9
Gastrectomy (distal)	2011	34 160	1774/5.2	709/2.1	208/0.6	451/1.3
	2012	38 750	2205/5.7	849/2.2	232/0.6	516/1.3
	2013	39 957	2450/6.1	892/2.2	239/0.6	542/1.4
	2014	38 584	2356/6.1	941/2.4	264/0.7	523/1.4
	2015	37 819	2325/6.1	851/2.3	222/0.6	452/1.2
	2016	36 852	2314/6.3	825/2.2	249/0.7	473/1.3
	2017	35 517	2445/6.9	859/2.4	253/0.7	437/1.2
	2018	33 988	2327/6.8	737/2.2	227/0.7	393/1.2
Total gastrectomy	2011	18 652	1716/9.2	634/3.4	177/0.9	427/2.3
	2012	21 122	2135/10.1	758/3.6	224/1.1	503/2.4
	2013	19 035	1831/9.6	642/3.4	169/0.9	428/2.2
	2014	19 071	1840/9.6	698/3.7	185/1.0	379/2.0
	2015	18 695	1907/10.2	654/3.5	178/1.0	387/2.1
	2016	17 670	1835/10.4	638/3.6	174/1.0	336/1.9
	2017	14 840	1702/11.5	514/3.5	161/1.1	293/2.0
	2018	13 223	1529/11.6	487/3.7	148/1.1	265/2.0
Right hemicolectomy	2011	17 890	1150/6.4	588/3.3	213/1.2	410/2.3
	2012	21 034	1470/7.0	677/3.2	263/1.3	471/2.2
	2013	21 814	1527/7.0	721/3.3	280/1.3	538/2.5
	2014	22 446	1544/6.9	771/3.4	287/1.3	530/2.4
	2015	22 850	1607/7.0	769/3.4	301/1.3	534/2.3
	2016	22 829	1510/6.6	791/3.5	253/1.1	449/2.0
	2017	22 543	1648/7.3	785/3.5	296/1.3	450/2.0
	2018	22 610	1679/7.4	740/3.3	276/1.2	424/1.9
Low anterior resection	2011	16 984	1616/9.5	1213/7.1	75/0.4	136/0.8
	2012	20 321	2092/10.3	1413/6.9	88/0.4	149/0.7
	2013	21 096	2059/9.8	1473/7.0	80/0.4	175/0.8
	2014	21 861	2098/9.6	1546/7.1	70/0.3	152/0.7
	2015	22 493	2210/9.8	1550/6.9	95/0.4	156/0.7
	2016	21 387	2306/10.8	1492/7.0	68/0.3	126/0.6
	2017	20 879	2376/11.4	1330/6.4	96/0.5	148/0.7
	2018	20 636	2454/11.9	1424/6.9	90/0.4	142/0.7
Hepatectomy (non‐lateral segments)	2011	7434	886/11.9	203/2.7	155/2.1	303/4.1
	2012	8239	1146/13.9	248/3.0	142/1.7	293/3.6
	2013	7937	1135/14.3	226/2.8	130/1.6	290/3.7
	2014	7666	1052/13.7	242/3.2	94/1.2	208/2.7
	2015	7439	1049/14.1	213/2.9	87/1.2	182/2.4
	2016	7610	1046/13.7	220/2.9	96/1.3	178/2.3
	2017	7698	1160/15.1	221/2.9	97/1.3	169/2.2
	2018	7192	1137/15.8	211/2.9	83/1.2	163/2.3
Pancreaticoduodenectomy	2011	8305	1285/15.5	299/3.6	97/1.2	238/2.9
	2012	9329	1654/17.7	365/3.9	137/1.5	281/3.0
	2013	10 068	1853/18.4	407/4.0	142/1.4	307/3.0
	2014	10 400	1847/17.8	374/3.6	111/1.1	267/2.6
	2015	10 576	2025/19.1	378/3.6	120/1.1	247/2.3
	2016	11 028	2242/20.3	393/3.6	98/0.9	232/2.1
	2017	11 580	2539/21.9	413/3.6	145/1.3	232/2.0
	2018	11 626	2716/23.4	402/3.5	111/1.0	204/1.8
Acute diffuse peritonitis surgery	2011	7753	2022/26.1	634/8.2	697/9.0	1096/14.1
	2012	9177	2456/26.8	685/7.5	785/8.6	1289/14.0
	2013	10 447	2652/25.4	786/7.5	861/8.2	1408/13.5
	2014	12 085	2966/24.5	937/7.8	927/7.7	1472/12.2
	2015	13 030	3126/24.0	1051/8.1	943/7.2	1551/11.9
	2016	13 981	3445/24.6	1068/7.6	1052/7.5	1572/11.2
	2017	14 423	3756/26.0	1125/7.8	1152/8.0	1575/10.9
	2018	14 835	3943/26.6	1183/8.0	1117/7.5	1617/10.9

^*^ Complications were defined by Clavien‐Dindo gradeⅢa‐Ⅴ.

**FIGURE 3:**
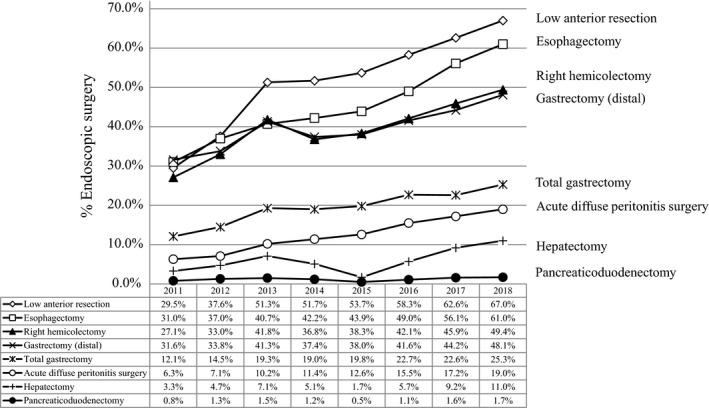
Annual changes in the percentage of surgeries carried out by endoscopic surgery: analysis of the eight major surgical procedures

An increase in the incidence of endoscopic surgery over time is shown in Figure 3. Endoscopic surgeries have been prevalent, especially in gastrointestinal procedures, with 67.0% for low anterior resection and 61.0% for esophagectomy in 2018. Even for acute diffuse peritonitis, laparoscopic surgery had been done in 19.0% of cases in 2018.

## 4 DISCUSSION

The National Clinical Database, a robust nationwide registry on surgical outcomes, shows the real status of Japanese surgical treatment. The purpose of NCD was to systematically gather clinical information, analyze these data for quality improvement, follow the best medical practices, and maintain a high standard of care for all people in Japan.

More than half of all patients who underwent surgical treatment in the gastroenterological field were senior patients aged 65 years or over. With the increase in the aging population, the rates of preoperative comorbidities such as diabetes mellitus or hypertension also increased. Age category was reported as a risk factor both for operative morbidity and mortality in all eight main procedures.^5^, ^6^, ^7^, ^8^, ^9^, ^10^, ^11^, ^12^, ^13^, ^14^, ^15^, ^16^, ^17^, ^18^, ^19^, ^20^ Although the annual rate of postoperative complications with C‐D classification of grade III or higher gradually increased over time, the postoperative mortality was kept at a low level. These results may be explained by the high participation rate of board‐certified surgeons in gastroenterological surgeries.^23^ The annual percentage of surgeries with participation of board‐certified surgeons in gastroenterological surgeries in the eight procedures gradually increased and the operative mortality was kept at a low level. Also, centralization of the surgical center may be important for improving surgical outcomes. The operative mortality rate after distal gastrectomy definitively decreased as both surgeon volume and hospital volume increased.^25^ After risk adjustment for surgeon and hospital volume and patient characteristics, hospital volume was significantly associated with low operative mortality. As for esophagectomy, high‐volume hospitals had a lower risk‐adjusted mortality rate compared with low‐volume hospitals.^26^ The factors causing these phenomena should be investigated for each procedure.

The rates of endoscopic surgery have been increasing year by year. This is the first report of the annual number of endoscopic surgeries in the selected 115 gastrointestinal operative procedures in the training curriculum for board‐certified surgeons in gastroenterology. Studies using the NCD data showed that the length of hospital stay was significantly shorter in patients who underwent endoscopic surgery for gastrectomy^27^, ^28^, ^29^, ^30^ and hepatectomy.^31^, ^32^ Laparoscopic liver resection has been safely developed with a low mortality and complication rates relative to open liver resection.^32^ Laparoscopic gastrectomy is also safe and feasible even for elderly patients^33^ or those with poor physical status, such as the American Society of Anesthesiologists physical status (ASA‐PS) class ≥3.^34^

A risk‐adjusted analysis based on nationwide data allows personnel to establish and provide feedback on the risks that patients face before undergoing a procedure.^21^ The risk calculator for all eight procedures is available on the websites of the hospitals that are part of the NCD. Standardized information on patient risk and predicted mortality can be reformulated as case reports and shared at conferences. In liver transplant recipients, real‐time risk models of postoperative morbidities and mortality at various perioperative time points were established.^35^ These real‐time risk models provide the expected risk of morbidities and mortality at any time point from pre‐, intra‐, and postoperative periods within 30 days after the surgery. With the availability of real‐time risk models of postoperative morbidity and mortality at each time point post‐surgery, the treatment team and caregivers might be encouraged to pay attention and possibly prevent or enhance recovery from specific morbidities and avoid mortality. These real‐time risk models may be applicable to other surgical procedures. The NCD also provides data on each facility's severity‐adjusted clinical performance (benchmark), which can be compared with national data.^21^, ^36^, ^37^ We can trace, periodically, where we are in the national standard, which will improve surgical care on an international basis.

Many valuable studies that use “big data” from the NCD have been published in succession. The NCD has contributed to evidence‐based medicine, to the accountability of medical professionals, and to quality assessment/improvement of surgery in Japan.

## DISCLOSURE

Conflict of interests: Arata Takahashi, Hiroyuki Yamamoto, and Hiroaki Miyata are affiliated with the Department of Healthcare Quality Assessment at the University of Tokyo. The department is a social collaboration department supported by grants from the National Clinical Database, Johnson & Johnson KK, and Nipro Co.
